# Knowledge, Attitudes, and Practices Associated with the Selection of Sweetened Ultra-Processed Foods and Their Importance in Oral Health

**DOI:** 10.3390/dj12080268

**Published:** 2024-08-20

**Authors:** María del Pilar Angarita-Díaz, Johao Alexander Colmenares-Pedraza, Valentina Agudelo-Sanchez, Juliana Alejandra Mora-Quila, Laura Sofia Rincón-Mejia

**Affiliations:** 1 Faculty of Dentistry, Campus of Villavicencio, Universidad Cooperativa de Colombia, Villavicencio 500001, Colombia; valentina.agudelos@campusucc.edu.co (V.A.-S.); juliana.mora@campusucc.edu.co (J.A.M.-Q.); laura.rinconm@campusucc.edu.co (L.S.R.-M.); 2 Food Security and Nutrition Dimension, Public Health Department, Villavicencio Health Secretariat, Villavicencio 500001, Colombia; jcolmenares@unillanos.edu.co

**Keywords:** food labelling, health knowledge, attitudes, practice, dietary sugars, oral health

## Abstract

Background: Reading nutritional labelling helps consumers select healthier food, thereby benefitting their oral health. The purpose of this study was to describe the knowledge, attitudes, and practices (KAPs) of parents and carers of children and preteens, associated with reading nutritional labelling, selecting sweetened ultra-processed foods, and their impact on oral health. Materials and Methods: This was a descriptive cross-sectional study in which a validated questionnaire was administered to 298 parents and caregivers of children aged 1–12 years from different districts in Villavicencio, Colombia. Participants’ scores, based on the number of correct answers, were used to classify their level of KAP as low, medium, or high. In addition, the frequency of responses, the KAP levels, and the median scores were analysed. Finally, associations were determined using the chi-square test. Results: Most participants reached a medium level in terms of knowledge (41.6%) and attitudes (49.3%) and a low level in terms of practices (43.3%). An association was found between participants’ level of KAPs and their socioeconomic and educational level (*p* < 0.05). Conclusions: The study findings reveal inadequate KAPs associated with nutritional labelling, adequate food selection, and the importance of oral health.

## 1. Introduction

With 41 million deaths each year, non-communicable diseases (NCDs) are among the leading causes of death worldwide [1]. Inadequate nutrition is a risk factor for the development of NCDs, such as cardiovascular diseases, diabetes mellitus, neoplasms, and dental caries [2]. In Colombia, the latest National Study for Oral Health (ENSAB IV) reported that 6.02% of 1-year-old children already have dental caries, with at least one tooth obturated, lost, or with untreated advanced caries. This percentage increases to 29.31% when incipient caries cases are included. In 3- and 5-year-old children, cases of caries increase by up to 47.10% and 62.10%, respectively, whereas this percentage reaches 54.16% among 12-year-old children [3].

Frequent consumption of fermentable carbohydrates, especially sugars such as sucrose, is one of the most important factors in the development of dental caries [4,5]. Saccharose favours the metabolism and multiplication of cariogenic bacteria, which alter the oral ecosystem and microbiota owing to high organic acid production [6,7]. These acids favour dental hydroxyapatite solubility and subsequent mineral loss [4,6,8]. As a means to tackle this issue, organisations such as the World Dental Federation have supported the guidelines for sugar intake proposed by the World Health Organization (WHO), inviting different oral health actors to coordinate actions aimed at decreasing sugar consumption to less than 5% of the daily total calorie intake or 25 g/day, reducing the risk of dental caries throughout life [9,10].

In countries such as Argentina, Brazil, Chile, Colombia, Costa Rica, Ecuador, Perú and Venezuela, mean sugar intake accounts for 20.1% of daily total energy levels [11]. Colombia is among the top three countries with the highest sugar consumption levels [11], and sugar is one of the most consumed ingredients among children aged <5 years and the fifth most consumed ingredient among those aged 5–12 years [12]. Ultra-processed foods, such as carbonated soft drinks, sweets, industrialised chocolate, flavoured drinks and desserts, which tend to have high sugar concentrations, are among the 40 most frequently consumed foods among children. Moreover, children aged <5 and 6–12 years eat sweets, on average, five and six times a week, respectively [12].

A lack of parents’ and caregivers’ awareness of healthy habits and the negative effects of this on oral health has been highlighted in different studies [13,14]. Knowledge, attitudes, and practices (KAPs) related to nutrition and its impact on oral health were also associated with the prevalence of diseases such as caries [15,16]. In addition, children’s free sugar consumption levels are influenced by their parents’ habits [17,18], lack of awareness regarding the importance of reading nutritional labelling, and/or a poor understanding of nutritional information [19].

Nutritional labelling on food packaging contains information about calories and other nutritional contents [20] and is intended to encourage healthy dietary habits and reduce non-communicable diseases associated with nutrition [21]. Front-of-package warning labelling also contributes to this, constituting a simple, practical, and effective way of indicating whether a food has high levels of sugar, fat, sodium or other nutrients that are critical to public health [22,23]. In Colombia, the use of front-of-package warning labelling was authorised for packaged foods with added salt/sodium, sugar, or fats at levels exceeding those established in the resolution [24,25].

Nutritional education is part of the intervention strategies used to improve oral health and promote healthy eating habits [26,27], which include consumers’ improved food selection as they read and understand nutritional labelling [28]. Before developing nutritional interventions, it is important to understand the context of the population of interest and how it relates to nutrition [29]. This study aimed to describe the KAPs of parents and carers of children and preteens in Villavicencio, Colombia, associated with reading nutritional labelling, selecting sweetened ultra-processed foods, and the importance of this selection in terms of oral health in 2022.

## 2. Materials and Methods

### 2.1. Study Design, Population, and Sample

A cross-sectional descriptive study was conducted from July to August 2022 in the city of Villavicencio, Colombia, authorised by the Ethics Subcommittee of Universidad Cooperativa de Colombia in August 2021 (No. BIO193). All methods were carried out in accordance with relevant guidelines and regulations.

The number of participants included in the sample was selected based on the number of households in Villavicencio, according to the 2018 DANE census (The National Administrative Department of Statistics) [30]. A representative sample of 298 parents or carers of children was selected, with a 90% confidence interval. Proportional Stratified sampling according to the population from each district was conducted in different safe neighbourhoods across the city. Participants were selected based on convenience sampling, on the street, in parks, sports centres, and outside educational institutions, shops, and churches. Selection criteria included being a parent or caregiver of children aged 1–12 years, having accepted to participate in the study by signing an informed consent form, and living in Villavicencio. This age group was chosen because children begin to receive a family diet from age one [31], and because it is at these ages that children are at high risk of developing dental caries [3]. Exclusion criteria included having an intellectual or cognitive disability or being illiterate.

### 2.2. Characteristics and Validity of the Survey

The validated questionnaire ‘KAP associated with sweetened ultra-processed foods selection and their Importance in Oral Health’ was applied, which consisted of four sections: (1) Socio-demographic data; (2) Knowledge of sugar and oral health, nutrition facts table, front-of-package warning labelling, nutritional labelling reading and its importance in oral health; (3) Attitudes regarding the importance of reading the nutrition facts table and reasons for reading it (if participants actually read it); and (4) Practices associated with reading nutritional labelling, reading frequency, correct ways of reading, sweet food consumption frequency and dentists’ recommendations (Appendix A).

Experts from different fields (nutrition, dentistry, public health, psychology, and epidemiology) participated in a focus group session to design the instrument. The instrument was then evaluated by seven experts (public health, health anthropology, social psychology, education, biology specialising in dental sciences, paediatric dentistry, and health promotion and prevention), and a high level of agreement between the evaluations was established. Sufficiency and pertinence were rated good (0.7–0.79), and clarity and concordance were rated excellent (0.81–1) for Kendall’s W (KW) values and a 95% confidence level. The questionnaire was then piloted with 20 parents or carers with the same characteristics as the study population. Finally, the instrument was validated with a sample of parents and carers of children between the ages of 1 and 12 from Villavicencio, calculated to be representative of the population. The internal consistency of the questionnaire was good (Kuder Richardson > 0.70), with a biserial index of 0.2–1 (very good) and a discrimination index of 0.4–1 (very high) and 0.3–0.39 (high) for most of the questions. In addition, the non-response index ranged from 0 to 0.29 (acceptable). The difficulty index ranged from 0.75 to 1 (very easy) for seven questions to 0.00–0.24 (very difficult) for five questions.

During questionnaire validation, three qualification levels were determined based on the number of correct answers. For the knowledge section, qualification was low for 0–1 correct answer, medium for 2 correct answers and high for 3–6 correct answers. Regarding the attitudes section, qualification was low for zero correct answers, medium for one correct answer and high for two correct answers. With respect to the practices section, qualification was low for 0–2 correct answers, medium for 3–4 correct answers and high for 5–12 correct answers.

### 2.3. Data Analysis

The SPSS 27.0 statistical package (IBM Corp., Armonk, NY, USA) was used to estimate the percentages of the sociodemographic characteristics, the answers to the questionnaire questions, and the level achieved in the KAP sections. The Kolmogorov–Smirnov test was used to determine the statistical normality of the data. Descriptive analysis was used to determine the number of correct answers, with median and interquartile range (IQR). In addition, the Chi-square test was used to determine a possible association between sociodemographic variables and the level of KAP achieved by the participants. A value of *p* < 0.05 was considered statistically significant for analysis.

## 3. Results

### 3.1. Demographic Characteristics of Participants

Most participants were female (79.5%, *n* = 237), were married or in a common-law relationship (54%, *n* = 161), had a higher education level (57%, *n* = 170), had employment as their main occupation (76.8%, *n* = 229), were from the lower socio-economic class (50%, *n* = 149), had only one or two children (91.9%, *n* = 274), and were aged 29–59 years (59.1%, *n* = 176) (Table 1). The median age was 32.0 and an interquartile range [IQR] of [24.0–32.0] years.

### 3.2. Knowledge of Nutritional Labelling and Its Importance in Oral Health

**Figure 1:**
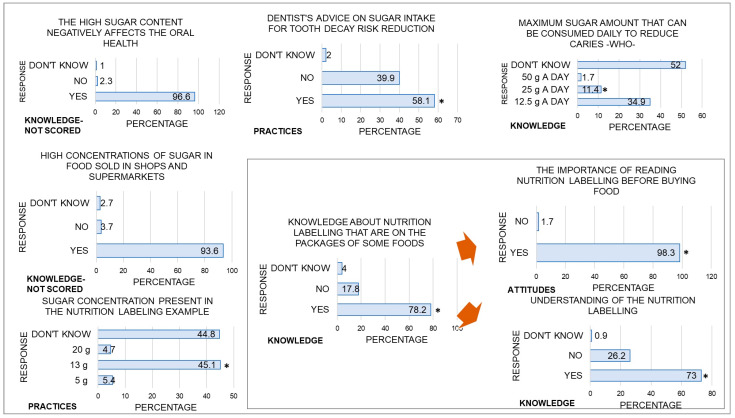

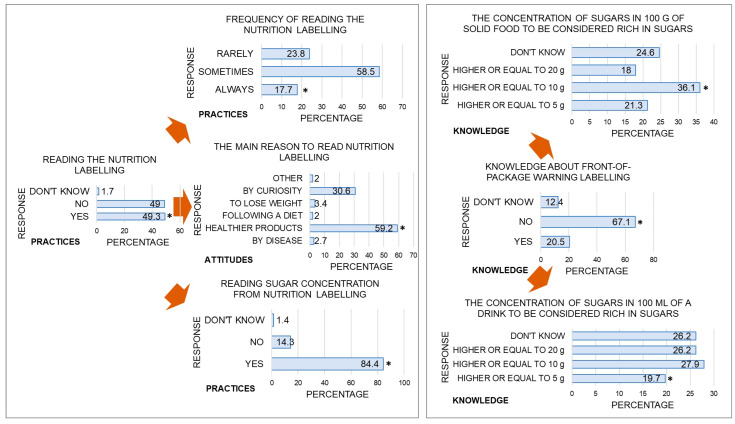
Percentage of responses selected by participants for the survey questions. * Correct answers. The orange arrows indicate the questions answered by participants when they had answered ‘yes’ to the prior question.

In the questions corresponding to the knowledge section, participants obtained correct answers with a median of 2.0 and an IQR of [1.0–2.0], and 41.6% (*n* = 124) of them reached a medium level in this section (Table 2). When asked about the high sugar content of foods sold in supermarkets and whether this was bad for health, more than 90% of participants answered ‘yes’. Most participants (78.2%) also said they were familiar with nutritional labelling. However, this percentage decreased when participants were asked if they were familiar with front-of-package warnings labelling (20.5%), and when asked about the maximum amount of sugar that can be consumed to avoid tooth decay (11.4%). Additionally, the amount of sugar that a solid or liquid food should contain to be considered high in sugar (36.1% and 19.7%, respectively). Finally, 73% of participants who said they were familiar with the Nutrition Facts Panel said they understood it (Figure 1).

### 3.3. Attitudes towards Nutritional Labelling and Their Importance in Oral Health

The attitudes section showed a median score of 1.0 and an IQR of [1.2–2.0], with 49.3% (*n* = 147) of participants scoring at an intermediate level (Table 2). In addition, most participants (98.3%) who answered ‘yes’ to the questions about their knowledge of nutrition labelling believed that reading this information was important. Finally, 59.2% of those who read labels said they did so to choose healthier products, while 30.6% said they did so out of curiosity (Fig 1).

### 3.4. Practices Associated with Nutritional Labelling and Their Importance in Oral Health

The median correct score for the practices section was 3.0 and an IQR [2.0–4.25], with many participants having a low level of understanding (43.3%, *n* = 129), followed by a medium level (31.9%, *n* = 95) (Table 2). Regarding the questions asked, most participants (58.1%) reported that they received advice from their dentists on the maximum amount of sugar children could eat to reduce caries. However, a significant proportion reported not receiving this information (39.9%). Furthermore, only 49.3% of participants answered ‘yes’ when asked if they read the nutrition table; when asked how often, many answered ‘sometimes’ (58.5%), followed by ‘rarely’ (23.8%). Finally, 84.4% of participants who read this information looked for the sugar content (Figure 1).

The most consumed solid and sweet ultra-processed foods among the children under the responsibility of the study participants were packaged biscuits, cakes, or sweetbread, and most participants (32.6%) reported a consumption frequency of more than once a month, followed by three times a week (25.2%). Additionally, 32.6% reported that their children ate sweets or candies one to three times a week, followed by once a month (25.2%). Regarding sweet drinks, most children rarely consumed soft drinks, artificial juice, or sweet-flavoured milk (34.2%, 24.2%, and 24.8%, respectively), although a significant percentage consumed them more than once a month (23.5%, 22.8%, and 16.4%, respectively) and even one to three times a week, once a day or more than once a day. Fruit-flavoured yoghurt is eaten by most children more than once a month (25.2%), rarely (24.8%), or one to three times a week (18.8%) (Table 3).

### 3.5. Factors Influencing Knowledge, Attitudes, and Practices Associated with Nutritional Labelling and Its Importance in Oral Health

A significant association between participants’ knowledge and variables such as socioeconomic level, educational level, and main occupation was found (*p* < 0.05). For attitudes, an association was found with socioeconomic level, educational level, and sex (*p* < 0.05). We also observed an association between practices and socioeconomic and educational variables (Table 4).

## 4. Discussion

Lifestyle changes have led to changes in dietary patterns, with a trend towards the consumption of low nutritional quality processed foods, and a low consumption of fruits, vegetables, and legumes. This influences the development of obesity and chronic diseases and it affects children’s social and emotional well-being and reduces their quality of life [32]. For this reason, the World Health Organization highlights good nutrition as part of food security to reduce the incidence of NCDs [33]. Nutrition is also important in the aetiology of dental caries, as oral hygiene and fluoride intake are inadequate in childhood, thus, reinforcing healthy eating habits is crucial in this age group [34].

The present study showed medium and low levels of KAPs among the parents and carers of children and preteens, associated with sweetened ultra-processed foods selection and their effect on oral health. The study also reveals key elements that could be used to educate the population on this topic and encourage positive attitudes and practices when selecting types of food. One of these elements is that although participants know that the foods sold in the supermarket are high in sugar, very few read the nutrition facts label, or they do so occasionally or rarely. This is due to factors that influence reading the nutritional labelling, such as flavour, habits, pressure exerted by the children to buy the product, time, lack of nutrition information, low educational level, finances, and poor understanding of how to read nutritional labelling [35,36,37].

Highlighting participants’ poor understanding of how to read nutritional labelling is the fact that most answered the study questions incorrectly, even though they claimed to understand the nutrition facts table and considered it important to read it. A study conducted in the United States found a higher percentage of participants that could correctly read three types of nutritional labelling in a sample of around 992 adults. However, the majority of the population in that study was younger and had a higher educational level [38]. Meanwhile, in a study conducted in China, only 22.1% of parents whose socio-demographic characteristics were similar to the ones in our study were able to understand the information presented in nutritional labelling [37]. This shows that socio-demographic factors must be considered when applying an educational strategy to improve KAP associated with nutritional education and nutritional labelling use.

As for the effects of socio-demographic factors, we found an association between having a low socioeconomic and educational level and achieving low KAPs levels, which is consistent with the findings of other studies [39,40,41]. This is because low-income individuals have a higher likelihood of adopting unhealthy attitudes and dietary habits as they have limited access to certain foods and to information on nutrition, thereby negatively affecting their nutrition literacy [39,40]. An additional factor is that fact that the importance attributed to nutritional quality decreases among individuals in difficult economic situations, while individuals with higher educational levels are more likely to obtain and understand the nutritional information they need and use it adequately when making dietary decisions [39,40,41]. In sum, education is one of the most significant determinants of health and nutrition literacy [40].

Consistent with the study conducted by Giro-Candanelo et al. in Spain in 2022, our study found that sex affected the attitude dimension, as men displayed a better attitude towards nutritional labelling [41]. Conversely, the study conducted by Liao and Yang in China in 2023 found that women had significantly better attitudes towards the need for and use of nutritional labelling, and this was associated with the fact that most of them had received nutritional training [42].

Another element that should be considered when applying an educational strategy to reading nutritional labelling is associated with front-of-package warning labelling. This type of labelling was implemented in Colombia in 2021 [24], which is why, in contrast to the participants in the study conducted in 2020 by Alaniz-Salinas and Castillo-Montes in Chile [43]. In that study, 98.7% of Chilean participants recognised warning labelling because it had been used since 2016 [44]. Although warning labelling is useful, other factors are more influential when buying foods. These include price, food category, lack of similar alternative foods, poor understanding, educational and economic levels, and preferences regarding flavour [45,46].

A preference for sweet flavours makes children more likely to eat high-calorie foods on a frequent basis, increasing the prevalence of caries [47]. In our study, most participants reported a low frequency of sweet, ultra-processed food consumption. However, some participants reported a fairly high frequency of consumption—three, four, or six times a week, and even once a day—of foods such as soft drinks, juice, flavoured milk, sugared cereal, biscuits, cakes, sweet bread, and sweets. A study conducted in Colombia in 2006 with a sample of 223 schoolchildren aged 5–12 years from low and middle socioeconomic levels found that processed and ultra-processed food consumption accounted for 34.4% of the daily total energy intake, suggesting decreased nutritional quality [48]. The authors of this study highlighted that these results support the evidence of a transition towards a dietary pattern characterised by higher consumption of much lower quality processed foods among children in Colombia [48].

In our study, some of the solid, ultra-processed foods most frequently consumed by participants’ children include biscuits, cakes or sweet bread, and sweets or candy, and most children ate these more than once a month. A study conducted with teenagers in Rio de Janeiro, Brazil, found that ultra-processed foods such as biscuits, sweets, and sweet drinks such as soft drinks, juice, and flavoured drinks were among the most frequently consumed products [49]. Another study conducted in Barbacena, Brazil, involving children aged 7–9 years attending a public school found that 38.6% of students ate this type of food more than three times a day [50]. This consumption frequency was higher than the frequency observed in our study, and this difference may be due to the fact that salty ultra-processed foods were included in the Brazil study, and because of the age differences between the children included in both studies and the method used to assess food intake.

Our study found a low frequency of consumption of sweet drinks, such as soft drinks and processed juices, as most participants reported a frequency of ‘rarely’, followed by ‘more than once a month’. These frequencies are not as high as the ones observed in the ENSIN (The National Nutritional Situation Survey of Colombia) 2010 data, which showed a consumption frequency of sweet drinks of 0.60 times a day in 81.8% of children aged between 5 and 10 years [51]. These values are higher than those reported by the National Survey on Children’s Health in the United States in 2021, where 57.1% of the population studied (children aged 1–5 years) reported having consumed a sugary drink at least once during the week before the survey [52]. In 2015, the ENSIN data showed a decrease in soft drink, tea, and soda consumption, with a frequency of 0.4 and 0.5 times a day (3.5 times during the week) in 76.1% of children aged 3–4 years and in 81.8% of children aged 5–12 years, respectively [12].

Fruit-flavoured yoghurt was the most frequently consumed sweet food, as it is considered to be healthy given its protein, calcium, and probiotic content. However, some brands produce yoghurts with high sugar concentrations, making them unhealthy products [52,53,54,55].

Dentists play an important role in oral health promotion and prevention, which includes increasing patients’ knowledge and awareness regarding healthy diets, especially focusing on reducing sugar consumption [10]. Nevertheless, 40% of our study participants reported not having received recommendations from their dentists on the maximum amount of sugar consumption to prevent caries. It is, therefore, important to consider WHO/FDI recommendations for incorporating nutritional education within training programmes aimed at health professionals so that they can effectively communicate this information to their patients [9,56].

Preventing NCDs and reducing their prevalence requires different strategies to promote appropriate habits, especially in the family environment, as this influences children’s eating behaviour through the availability of healthy foods. Such strategies should include educational aspects and address KAP associated with the risk of disease in the population, such as eating large quantities of sugary foods [57,58]. Promoting public health policies that encourage healthy eating, ensuring transparent food labelling, and limiting the marketing and availability of foods high in fat, salt, and sugar are also important [9,10].

Limitations of this study include the type of sampling used for selecting participants, which was non-probabilistic. This is due to the fact that we did not have the resources, or the time required to address the entire population, which also implied entering unsafe parts of the city, putting the data-collecting personnel at risk. Another limitation involved the fact that although the 1 to 12 years age range was selected based on the available evidence regarding the life cycle with a higher predisposition to the risk of cavities, dietary patterns and the ability and autonomy to choose foods may vary within the same group. Therefore, this could affect the estimate of the prevalence of consumption of products with high sugar content.

## 5. Conclusions

The population surveyed in Villavicencio exhibited a medium level of knowledge and attitudes and a low level of practices associated with oral health and the selection of sweet foods. This highlights the need for the implementation of strategies aimed at increasing knowledge and attitude levels and promoting healthy habits associated with the selection and consumption of low-sugar foods. This approach may help reduce the prevalence of caries.

## Figures and Tables

**Table 1:** Socio-demographic characteristics of study participants.

Characteristic	Percentage (%)
Sex	
Male	20.5 (*n* = 61)
Female	79.5 (*n* = 237)
Marital Status	
Single	41.6 (*n* = 124)
Married or cohabitation	54 (*n* = 161)
Divorced/Widowed	4.4 (*n* = 13)
Education levels	
Basic education	2.3 (*n* = 7)
Secondary-High school education	40.6 (*n* = 121)
Higher education	57 (*n* = 170)
Main occupation	
Studying	3.4 (*n* = 10)
Working	76.8 (*n* = 229)
Homemaker	16.4 (*n* = 49)
Unemployed/pensioner	3.4 (*n* = 10)
Socioeconomic status	
Lower	50 (*n* = 149)
Middle	49 (*n* = 146)
Upper	1 (*n* = 3)
Age group	
18–28	37.2 (*n* = 111)
29–59	59.1 (*n* = 176)
>60	3.7 (*n* = 11)
Number of children	
1 to 2	91.9 (*n* = 274)
3 to 4	6.4 (*n* = 19)
5 or more	1.7 (*n* = 5)

For this analysis, marital status, socioeconomic status, educational level, and main occupation were recategorized.

**Table 2:** Knowledge, attitudes and practices scores and levels associated with nutritional labelling and its importance in oral health.

Level by Dimension	Percentage (%)
Knowledge	
Low (0–1 correct answers)	37.9 (*n* = 113)
Medium (2 correct answers)	41.6 (*n* = 124)
High (3–6 correct answers)	20.5 (*n* = 61)
Score [median (IQR)]:	2.0 (1.0–2.0)
Attitudes	
Low (0 correct answers)	22.5 (*n* = 67)
Medium (1 correct answer)	49.3 (*n* = 147)
High (2 correct answers)	28.2 (*n* = 84)
Score [median (IQR)]:	1.0 (1.0–2.0)
Practices	
Low (0–2 correct answers)	43.3 (*n* = 129)
Medium (3–4 correct answers)	31.9 (*n* = 95)
High (5–12 correct answers)	24.8 (*n* = 74)
Score [median (IQR)]:	3.0 (2.0–4.25)

**Table 3:** Frequency of sweet ultra-processed foods consumption among participants’ children or teenagers.

Frequency	Sweet Ultra-Processed Foods Consumption				
	Soft Drinks, Tea and Soda	Juice Packed in a Carton, Bottle or Bag	Energy Drinks	Sugared and Flavoured Milk	Fruit-Flavoured Yogurt
	Percentage (%)				
Never	8.7	9.7	96.0	34.6	9.4
Rarely	34.2	24.2	2.5	24.8	24.8
Once a month	15.1	18.1	1.5	13.8	13.1
More than once a month	23.5	22.8	0	16.4	25.2
One to three times a week	10.4	13.4	0	5.0	18.8
Four to six times a week	3.4	6.4	0	3.4	5.4
Once a day	4.7	4.4	0	1.7	3.0
More than once a day	0	1.0	0	0.3	0.3
Frequency	Sweet ultra-processed foods consumption				
	Soft drinks, tea and soda	Juice packed in a carton, bottle or bag	Energy drinks	Sugared and flavoured milk	Fruit-flavoured yogurt
	Percentage (%)				
Never	78.9	1.0	33.6	3.0	76.5
Rarely	11.0	8.6	28.9	16.4	7.0
Once a month	4.4	12.8	9.1	6.4	5.4
More than once a month	3.7	32.6	15.1	25.2	4.4
One to three times a week	1.0	25.2	9.1	32.6	3.0
Four to six times a week	0.7	8.7	3.4	7.4	2.0
Once a day	0.3	10.4	1.0	7.7	0.7
More than once a day	0	0.7	0	1.3	1.0

**Table 4:** Factors influencing knowledge, attitudes, and practices levels.

Socio-Demographic Variables	Level by Dimension			
	Low Level	Medium Level	High Level	*p*-Values ^¥^
	Percentage (%)			
Socioeconomic level	Knowledge			
Low	23.8%	18.8%	7.4%	0.002 **
Medium	14.1%	22.5%	12.4%	
High	0%	0.3%	0.7%	
Main occupation	Knowledge			
Student	0.7%	1%	1.7%	0.035 *
Employed	27.9%	32.6%	16.4%	
House chores	8.4%	6.4%	1.7%	
Unemployed/ Pensioned	1%	1.7%	0.7%	
Educational level	Knowledge			
Basic education	2.0%	0.3%	0%	<0.001 ***
High school education	22.1%	15.8%	2.7%	
Higher education	13.8%	25.5%	17.8%	
Sex	Attitudes			
Male	2.7%	13.1%	4.7%	0.027 *
Female	19.9%	36%	23.6%	
Socioeconomic level	Attitudes			
Low	14.4	8.1	0	0.025 *
Medium	22.8	25.8	0.7	
High	12.8	15.1	0.3	
Educational level	Attitudes			
Basic education	1.7%	0.7%	0%	<0.001 ***
High school education	12.8%	19.1%	8.7%	
Higher education	8.1%	29.5%	19.5%	
Socioeconomic level	Practices			
Low	24.5	15.8	9.7	0.018 *
Medium	18.5	15.8	14.8	
High	0.3	0.3	0.3	
Educational level	Practices			
Basic education	1.7%	0.3%	0.3%	<0.001 ***
High school education	22.1%	13.4%	5.0%	
Higher education	19.5%	18.1%	19.5%	

This table shows only the socio-demographic variables significantly associated with participants’ KAP levels. ^¥^ Analysis performed with the Pearson’s Chi-square test with the linear-by-linear association model. * *p* < 0.05, ** *p* < 0.01, *** *p* < 0.001.

## Data Availability

All data generated or analysed during this study are included in this published article.

## Appendix A. Questionnaire to Determine Knowledge, Attitude and Practice Associated with the Selection of Sweetened Ultra-Processed Foods and Their Importance in Oral Health

**Socio-demographic data**

1. What is your sex? Male/Female/Other
2. What is your marital status? Single/Married/Common-Law Marriage/Divorced/Widowed/Separated
3. What is the highest level of education you have achieved? None/Primary school (incomplete)/Primary school (complete)/High school (incomplete)/High school (complete)/One or more years of technical or technological education/One or more years of university/University (complete)/Graduate studies
4. What is your main occupation? Student/Employed/Student and Employed/House chores/Unemployed/Disabled/Permanently disabled/Pensioned/Don’t know-No opinion
5. What is your socioeconomic stratum? Stratum 0/Stratum 1/Stratum 3/Stratum 4/Stratum 5/Stratum 6
6. What is your age?
7. Where do you live permanently?
8. Please write the total number of children aged 1–12 years that you are responsible for.

**Knowledge, Attitudes and Practices (*Scored question)**

9. Do foods with high sugar content negatively affect the oral health of your child/children? Yes/No/Don’t know-No opinion
10. *According to the World Health Organization and World dental Federation, what is the maximum sugar amount that can be consumed daily to reduce the occurrence of caries? 12.5 g a day (almost three teaspoons)/25 g a day (5 teaspoons)/50 g a day (5 teaspoons)/Don’t know-No opinion
11. *Are you familiar with the nutrition facts tables that are on the packages of some foods? Yes/No/Don’t know-No opinion
12. *Do you believe it is important to read the nutrition facts tables before buying food? Yes/No/Don’t know-No opinion
13. *Do you understand the information presented in the nutrition facts tables? Yes/No/Don’t know-No opinion
14. *Do you read the nutrition facts tables on the packages of some foods? Yes/No/Don’t know-No opinion
15. *How often do you read the nutrition facts tables on the packages of the products you find in the supermarket? Always/Sometimes/Rarely/Never/Don’t know-No opinion
16. *What is the main reason for you to read the nutrition facts table? I have a disease/To choose healthier products/Following a diet/To lose weight/Out of curiosity/Other
17. *Do you check the amount of sugar when you read the nutrition facts table? Yes/No/Don’t know-No opinion
18. Do you believe that the sugar levels of the foods sold in supermarkets and shops are higher than the recommended ones? Yes/No/Don’t know-No opinion
19. *Are you familiar with front-of-package warning labelling? Yes/No/Don’t know-No opinion
20. *How much sugar should a solid food, such as biscuits, have in order for it to be considered high in sugar? Higher or equal to 5 g in 100 g (one teaspoon)/Higher or equal to 10 g in 100 g (two teaspoons)/Higher or equal to 20 g in 100 g (four teaspoons)/Don’t know-No opinion
21. *How much sugar should a drink, such as a soft drink, have in order to be considered high in sugar? Higher or equal to 5 g in 100 mL (one teaspoon)/Higher or equal to 10 g in 100 mL (two teaspoons)/Higher or equal to 20 g in 100 mL (four teaspoons)/Don’t know-No opinion
22. *Has your dentist provided you with recommendations on the maximum amount of sugar that your child should eat to reduce the risk of caries? Yes/No/Don’t know-No opinion
23. *The following figure shows the nutritional information of a dairy product. According to the following nutrition facts table, how many grams of sugar are consumed for every 100 g of food eaten? 26 g (almost five teaspoons of sugar)/17.4 g (almost five teaspoons of sugar)/5.6 g (almost five teaspoons of sugar)/Don’t know-No opinion
24. Please answer the following question based on the eating habits of the 1–12-year-old child that is under your responsibility. If there is more than one child of that age, please answer the question based on the youngest child’s habits.

How many times has the child eaten the following foods over the last month? There are 9 options: Never/Rarely/Once a month/More than once a month/One to three times a week/Four to six times a week/Once a day/More than once a day/Don’t know-No opinion

- *Soft drinks, tea and soda (powder, carton or bottle, except for diet drinks)
- Energy drinks (such as Vive 100, Monster, Peak and Gatorade)
- *Juice packed in a carton, bottle or bag
- *Packages of sweet biscuits, cakes or sweet bread
- *Sugared cereal ready for breakfast (such as sugared cornflakes)
- Compote or baby food packed in cartons or bottles
- Fruit-flavoured yogurt
- *Other types of sweet yogurt (such as Greek yogurt and Kefir)
- *Sugared and flavoured milk
- *Sweets or candy (sweets, lollipop, chocolate, jelly gums and mints)
